# Influence of weight-bearing on the 3D movement of lumbar facet joints in the sitting position

**DOI:** 10.1186/s12891-023-06698-y

**Published:** 2023-07-10

**Authors:** Ye Han, Xiaodong Wang, Jianzhong Wang, Shaosong Sun, Xi Xia, Jing Wang, Jun Miao

**Affiliations:** 1 Department of Orthopedics, The Affiliated Hospital of Hebei University, Hebei, China; 2 Department of Orthopedics, Baoding First Central Hospital, Hebei, China; 3 Department of Orthopedics, Gaoyang County Hospital, Hebei, China; 4 Department of Orthopedics, Tianjin Hospital of Tianjin University, Tianjin, China

**Keywords:** Weight-bearing, Lumbar facet joints, Sitting, Lumbar, DFIS

## Abstract

**Objective:**

To analyze the motion characteristics of lumbar facet joints and to observe the effect of weight-bearing on lumbar facet joints in the sitting position.

**Methods:**

Ten normal subjects (5 males and 5 females) were recruited and scanned by CT, and their lumbar 3D models were reconstructed by software. The images of flexion and extension of lumbar facet joints in the sitting position were collected without weight-bearing and weight-bearing 10 kg, and the 2D model was constructed by software. The 2D-3D model was matched to restore the flexion and extension motion changes of the subjects’ lumbar spine in the sitting position. Coordinates were established in the middle of the vertebral body and copied to the facet joints. Measure and record the lumbar facet joint movement distance through coordinate system. The relevant data of facet joints were collected.

**Results:**

In the L3/4 segment, after weight loading, the displacement of the left facet joint in the X axis became larger, while that in the Y axis and Z axis decreased. The displacement of the right facet joint in the X axis and Y axis increased, and the Z axis displacement decreased. The rotation angle of the bilateral facet joints also decreased. In the L4/5 segment, after loading, the displacements of the X, Y, and Z axis displacements of both sides increase, while the rotation angles of α and β increase, while the rotation angle of γ decreases. In the L5/S1 segment, the displacements of the X, Y, and Z axes on the left side decrease. The displacement of the X and Y axes on the right side decreases, while the displacement on the Z axis increases. The rotation angles of α and γ increase, and the rotation angle of the β axis decreases.

**Conclusion:**

When sitting, the flexion and extension distance and rotational displacement of lumbar facet joints are not affected by weight-bearing. In addition, there is asymmetry in the movement of the left and right facet joints, and weight bearing has no effect on the asymmetry of the motion.

## Introduction

Sitting posture is the most commonly used posture in human daily work and life. When sitting, the load on the lumbar vertebrae increases, and low back pain is more likely to occur [1–5]. Some previous studies have suggested that lumbar facet joints may be a potential source of low back pain [6–11], and facet joints degeneration can also accelerate lumbar disc degeneration.

Evaluating the effect of weight-bearing on lumbar facet joints movement in the sitting position can improve people’s understanding of lumbar facet joints movement. Kinematic parameters can also be used as a baseline to evaluate the effects of degenerative changes and surgery on facet joints, which can be used to prevent diseases and improve surgical techniques. However, the current research lacks data in this field. Therefore, we conducted related experiments to measure the effect of weight-bearing on lumbar facet joints in the sitting position.

At present, there are several methods used to measure the motion of lumbar facet joints, including imaging measurements [12, 13], finite element analysis techniques [14, 15], and dynamic system capture [16, 17]. However, due to the limitation of technical methods, most previous studies are based on a two-dimensional level, and there are some defects, such as low measurement accuracy. In our experiment, the Dual Fluoroscopic Image System (DFIS) combined with the CT technique was used to measure the flexion and extension of lumbar facet joints at the 3D level. This method has high accuracy, the translation error of reproducing spinal motion in the human body is less than 0.3 mm, and the rotation angle is less than 0.7° [18, 19]. It can be used to measure the motion of the lumbar facet joints accurately.

## Methods

### Participant recruitment

In this study, 10 healthy volunteers (5 males and 5 females) were recruited, 32 ± 4.29 years old, body mass index (BMI) 18.5–25.

#### Inclusion Criteria

(1) volunteers aged 20–50 years; (2) without chronic diseases such as cardiovascular, liver, or kidney diseases; (3) without abnormal lumbar and physical radiology examinations; (4) with a T value in bone density between − 1 and 1.

#### Exclusion criteria

(1) previous history of lumbar surgery and lumbar trauma; (2) spinal diseases, such as idiopathic scoliosis, human disease and other diseases that cause lumbar deformities; (3) pregnancy; (4) severe osteoporosis and other diseases that may affect the results of the test.

The study was conducted in accordance with the Helsinki Declaration (revised in 2013). We explained the research content to all the subjects and signed the informed consent form. This study was approved by the Research Ethics Committee of Tianjin Hospital of Tianjin University.

### Modelling technique

**Fig. 1:**
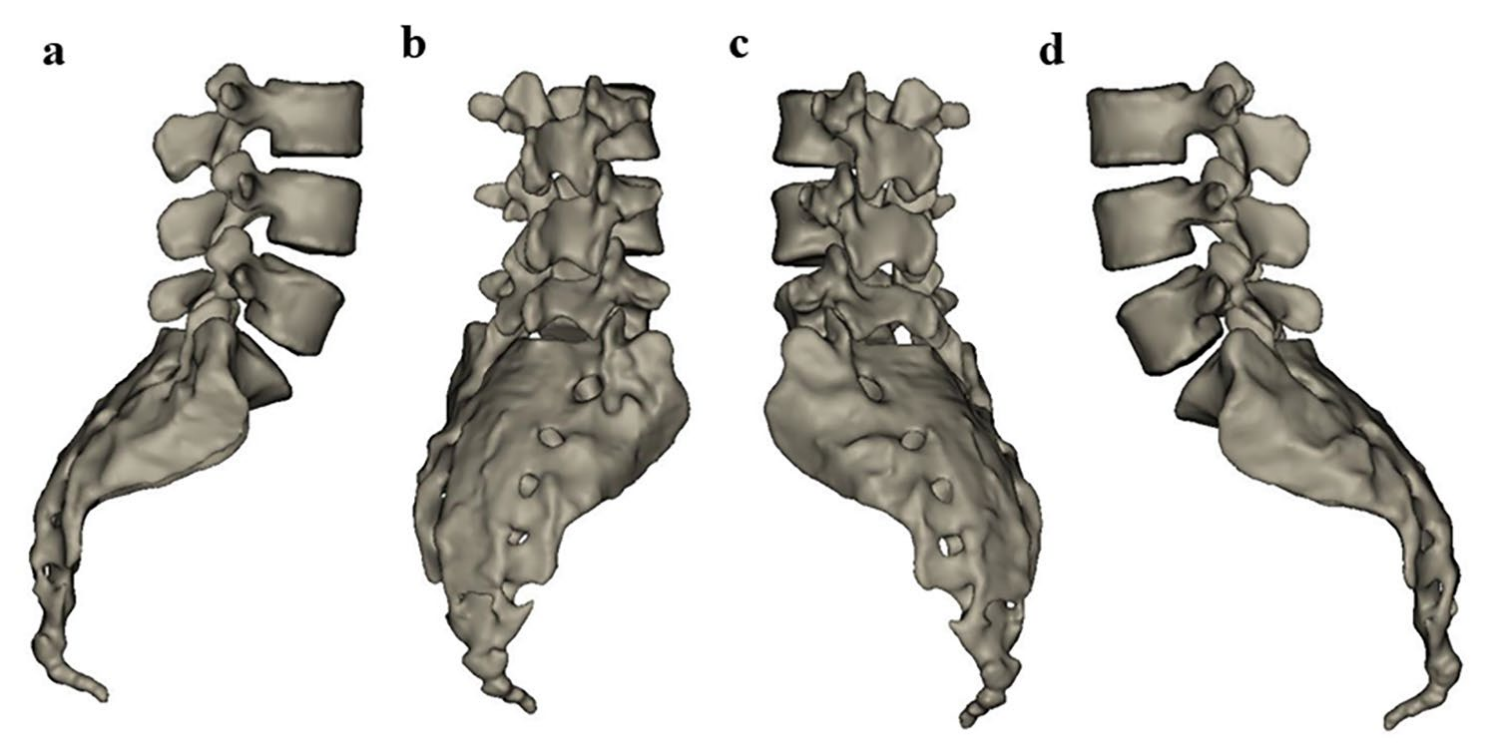
Lumbar vertebra model reconstructed by Mimics 19.0

The subjects completed CT scanning in the supine position with a slice thickness of 0.625 mm and a resolution of 512 pixels×512 pixels. The obtained image data were imported into Materialise Interactive Medical Image Control System software (version 19.0, Materialise NV, Belgium). By selecting a special bone threshold, the bone model was extracted, and the lumbar 3D model was reconstructed. (Fig. 1).

### Establishment of the dual fluoroscopic image system (DFIS)

**Fig. 2:**
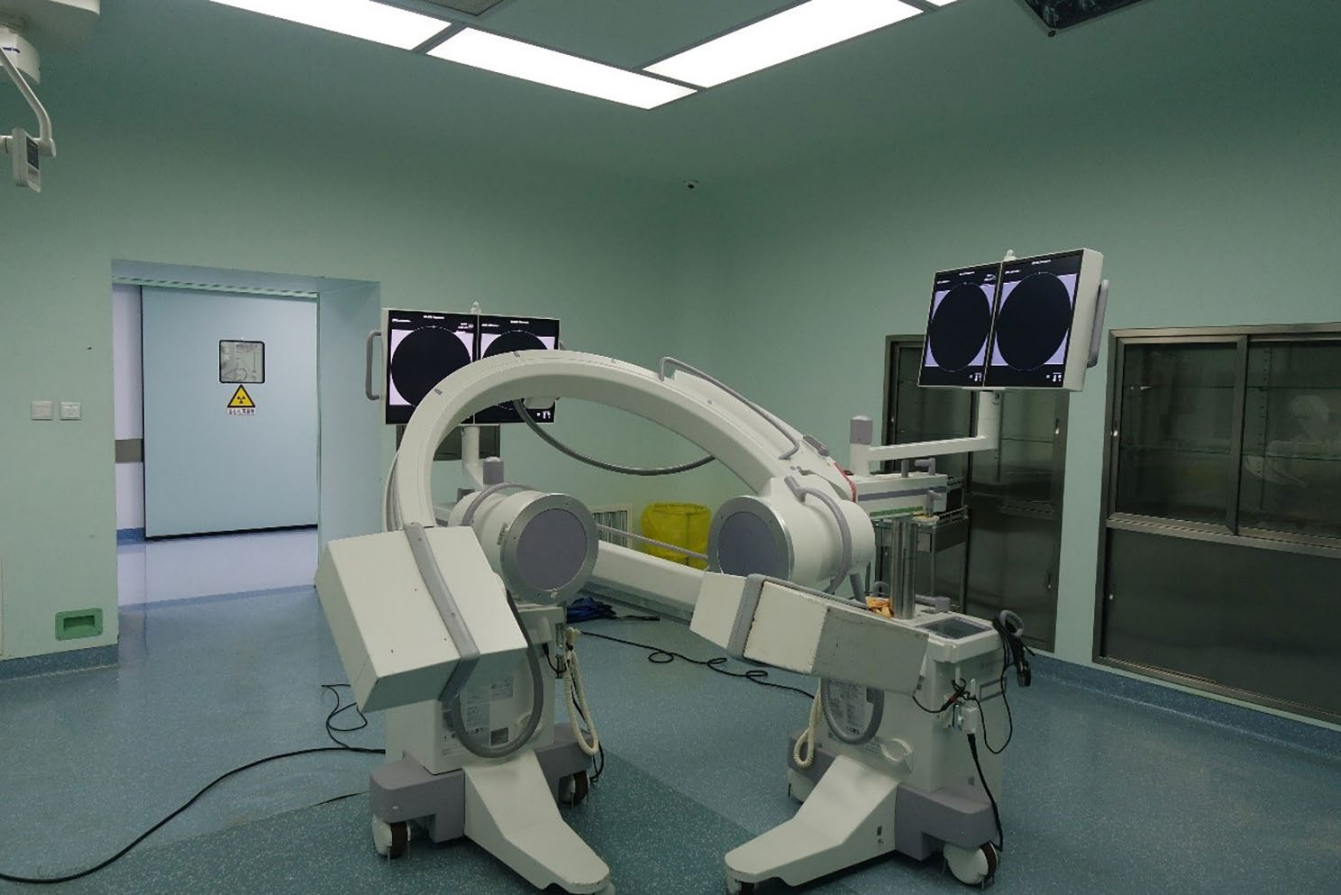
Dual fluoroscopic imaging system: The DFIS system was composed of two mobile X-ray machines placed perpendicular to each other

**Fig. 3:**
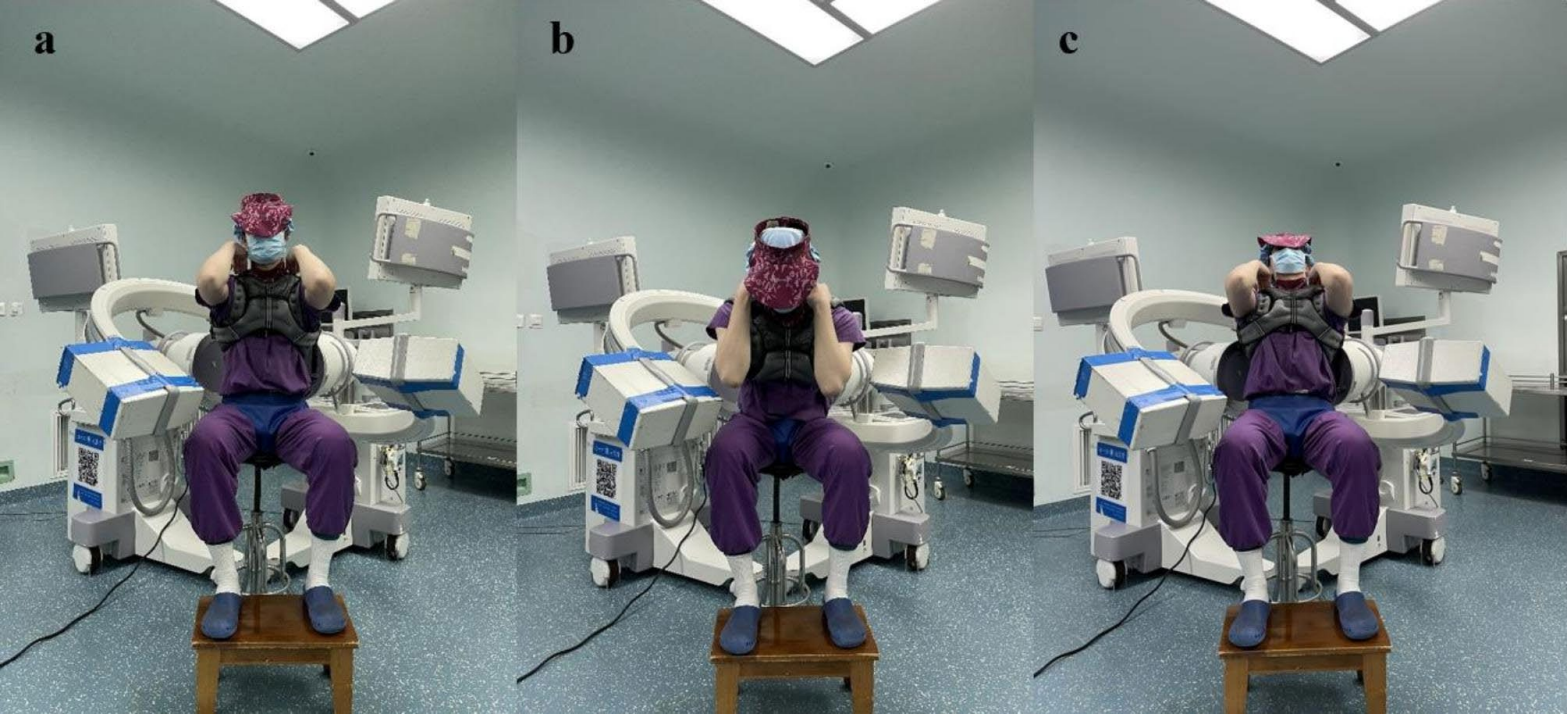
Participant subject sits in a height-adjustable seat and performs maximal flexion and extension movements with wear-bearing 10 kg. **(a)** Neutral position **(b)** Flexion **(c)** Extension

Two mobile C-arm machines are placed perpendicular to each other and debugged to form a DFIS system (Fig. 2). The subjects sat on a stool with adjustable height and adjusted according to their height. The subjects’ pelvis was fixed, keeping their thighs parallel and their calves perpendicular to the ground. Place both upper limbs on the shoulders and ensure that the lumbar vertebrae are the C-arm’s projection center and are in the image acquisition area. The subjects performed three movements: the neutral position, flexion position and extension position, and the movement maintained the maximum amplitude. X-ray (30 frames per second, 8 milliseconds pulse width, 1280 pixels×1024 pixels) lasted 1 s at each position, and a clear lumbar X-ray film was obtained. Then, the subjects carried a special 10 kg weight-bearing device, maintained the 30 min, and completed the X-ray collection of the same action again. The image acquisition process is directed by two spinal surgeons to ensure the accuracy of the action (Fig. 3). The obtained X-ray images are saved in DICOM format, and then image processing is carried out to remove diffraction.

### Establish a coordinate system

**Fig. 4:**
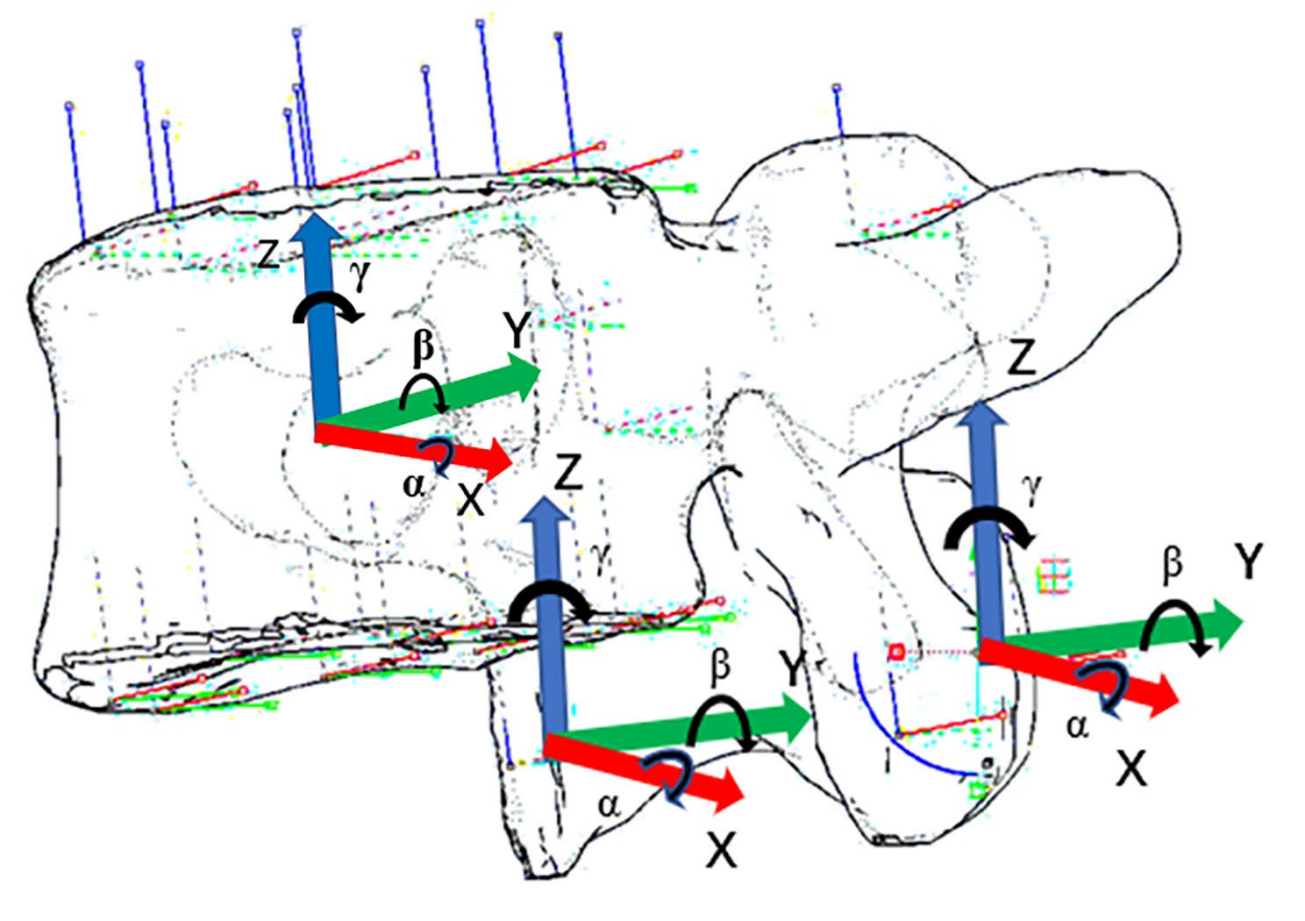
Established coordinate system, which establishes coordinates at facet, spinous process and other positions

The center point of the vertebral body was selected to establish a Cartesian coordinate system, which was divided into three axes: X axis (red), Y axis (green) and Z axis (blue). The X axis was the horizontal line to the left on the coronal plane, the Y axis was the horizontal line pointing back on the sagittal plane, and the Z axis was the vertical line pointing to the head on the sagittal plane. The displacements along each axis are recorded as x, y, and z. The clockwise rotation angles around the three axes of X, Y and Z are α, β and γ, respectively. The displacement consistent with the direction of the arrow is recorded as a positive number, and the opposite direction is recorded as a negative number. The clockwise rotation is recorded as a positive number, and the counterclockwise rotation is recorded as a negative number. The unit of displacement is expressed in mm, while the unit of rotation is expressed in °. The established coordinate system was copied at the midpoint of the inferior articular process of the cephalic vertebral body and the midpoint of the superior articular process of the caudal vertebral body, which was used to measure the displacement distance and rotation angle of the facet joints (Fig. 4).

### Reproduce the motion of lumbar facet joints

**Fig. 5:**
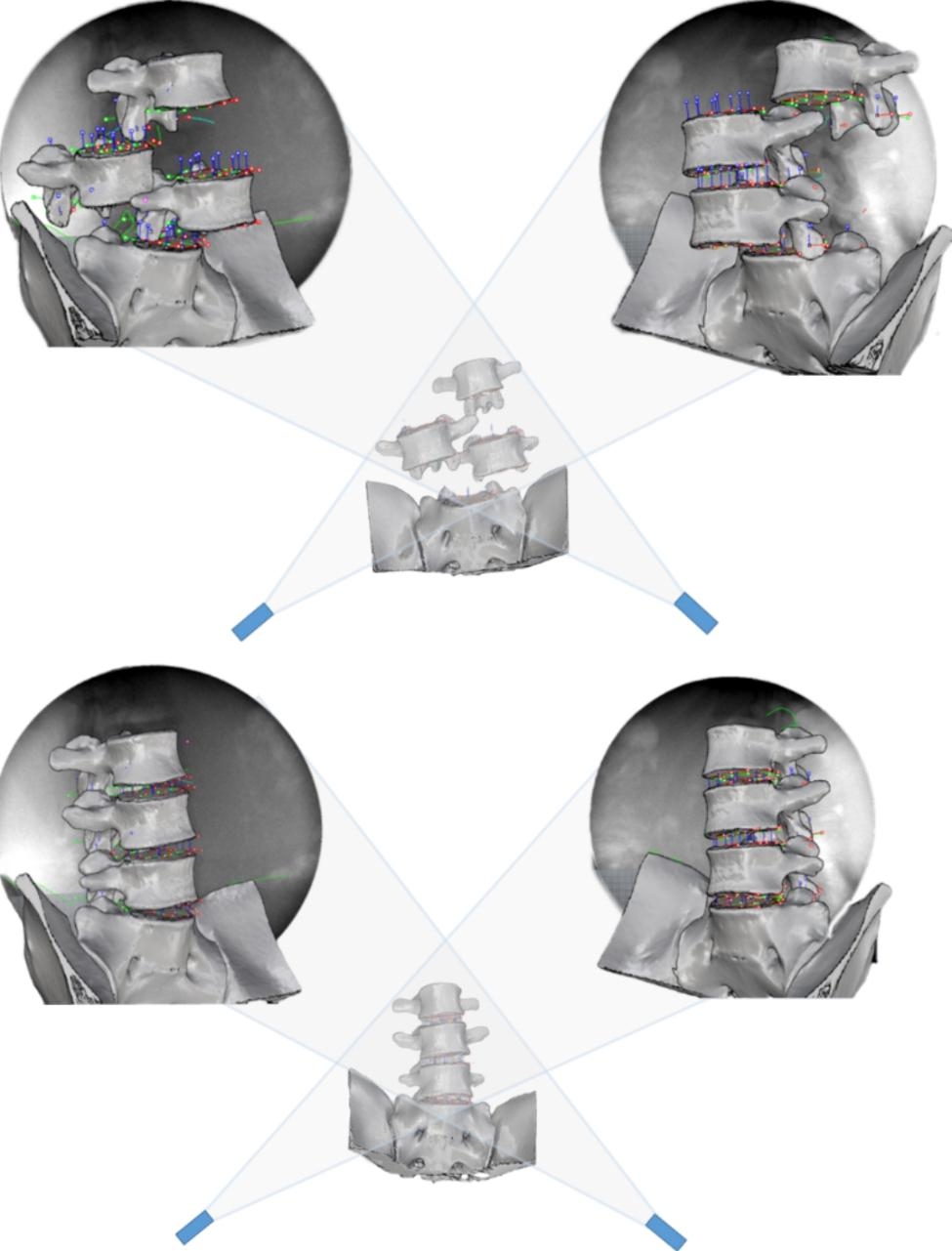
Spine vertebral bodies were adjusted in Rhinoceros software to match the 2D background and 3D model to restore vertebral body position in various postures

The modified X-ray images were imported into Rhinoceros software (version 5.0, Robert McNeel & Associates, United States). According to the method of Li et al. [20], the X-ray emission source and receiver were simulated in the software. The received image is used as the background image in the software, and the relevant tools are used to outline the vertebral body, lumbar facet joints, spinous process and other anatomical contours in the background image and complete the 2D modelling of the lumbar model. The 3D model built by CT is imported into the modelling software. According to the anatomical structure of lumbar vertebrae, the position of each vertebral body is adjusted so that it completely overlaps with the anatomical outline of the background, and the position matching of vertebrae with different movements of 2D-3D can be completed. The motion state of lumbar vertebrae in different positions (neutral position, flexion position, extension position) can be restored (Fig. 5).

#### Data measurement of the three-dimensional lumbar model

The facet joint coordinates in the neutral position of the spine were set as the origin, and then the displacement distance and rotation angle of the facet joint during the maximum flexion and extension activities of the spine were measured. Through the change in the relative position of the lumbar facet joint, the position change data of the corresponding motion were obtained. That is, the position comparison of L4 superior facet joint and L3 lower facet joint, L5 superior facet joint and L4 lower facet joint position comparison, S1 superior facet joint and L5 lower facet joint position comparison. The motion characteristics of lumbar facet joints in the sitting position were studied by comparing the data of flexion-extension without weight-bearing and flexion-extension during weight-bearing 10 kg.

### Data statistics and analysis

Statistical analysis was performed using SPSS version 26.0 (IBM, Armonk, NY, USA). Two-way repeated-measures ANOVA was used to compare the range of motion of the lumbar facet joints at the L3/4, L4/5 and L5/S1 vertebral levels under weightbearing conditions. Kinematics was the dependent variable, and weight bearing and vertebral body level were the independent variables. Data were tested by the Kolmogorov – Smirnov test and found to follow a normal distribution. A paired t test was used to compare the difference in bilateral facet joint displacement. Statistical significance was set at P < 0.05, with continuous variables expressed as X ± S.**Results**.

### The L3-4 segment

When the subjects were in the sitting position, the flexion and extension movement of the left facet joint was 0.00 ± 1.01 mm in the X axis, the Y axis was − 0.29 mm ± 1.14 mm, the Z axis was − 4.65 ± 1.99 mm, and the right facet joint was − 0.12 mm ± 0.31 mm in the X axis, the Y axis was-3.2 mm ± 0.82 mm, the Z axis was − 0.19 mm ± 0.36 mm, The rotation angle of facet joint was − 6.00 ± 4.49 °in α angle, 2.30 ± 2.90 °in β angle and 0.74 ± 1.57 °in γ angle.

When the subjects moved flexion and extension with weight bearing 10 kg in the sitting position, the displacement of the right facet joint in the X axis was 0.28 ± 0.05 mm, the Y axis was 0.03 ± 0.24 mm, the Z axis was − 2.72 ± 0.67 mm, the right facet joint was 0.23 ± 0.03 mm, the Y axis was 0.37 ± 0.21 mm, and the Z axis was − 1.26 ± 0.69 mm. The rotation angle of the facet joint was − 3.80 ± 1.14° in the α angle, 1.28 ± 0.68° in the β angle and − 0.27 ± 0.53° in the γ angle. There was no significant difference between 0 and 10 kg.

### The L4-5 segment

When the subjects were in the sitting position, the displacement of the left facet joint was 0.45 ± 0.21 mm in the X axis, the Y axis was − 0.41 ± 0.32 mm, and the Z axis was − 1.89 ± 0.95 mm. The displacement of the right facet joint was 0.77 ± 0.31 mm, the Y axis was 0.15 ± 0.51 mm, and the Z axis was − 1.72 ± 0.95 mm. The rotation angle of the facet joint was − 3.5 ± 1.66° in the α angle, 0.16 ± 0.65° in the β angle and − 0.94 ± 0.89° in the γ angle.

When the subjects were in the sitting position, the flexion and extension movement of the left facet joint was 0.66 ± 0.19 mm on the X axis, the Y axis was − 0.97 ± 0.35 mm, and the Z axis was − 2.51 ± 0.56 mm. The displacement of the right facet joint was 1.02 ± 0.26 mm in the X axis. The Y-axis was − 0.67 ± 0.14 mm, and the Z axis was − 2.55 ± 0.48 mm. The rotation angle of the facet joint was − 5.96 ± 1.05° in the α angle, 0.58 ± 0.47° in the β angle and − 0.55 ± 1.03° in the γ angle. There was no significant difference between 0 and 10 kg.

### The L5-S1 segment

When the subjects moved flexion and extension with weight bearing 0 kg in the sitting position, the displacement of the left facet joint in the X axis was 1.43 ± 0.88 mm, the Y axis was − 1.55 ± 1.32 mm, and the Z axis was 0.81 ± 0.72 mm. The displacement of the right facet joint was 1.40 ± 0.88 mm in the X axis, the Y axis was − 0.51 ± 1.07 mm, and the Z axis was 0.31 ± 0.53 mm. The rotation angle of the facet joint was 0.97 ± 1.31° in the α angle, -0.36 ± 0.55° in the β angle and − 1.12 ± 0.90° in the γ angle.

When the subjects were in the sitting position, the flexion and extension movement of the left facet joint was − 0.89 ± 0.38 mm in the X axis, the Y axis was 0.69 ± 0.71 mm, and the Z axis was 0.41 ± 0.67 mm. The displacement of the right facet joint in the X-axis was − 0.77 ± 0.40 mm in the X axis, the Y axis was − 0.33 ± 0.54 mm, and the Z axis was 0.92 ± 0.94 mm. The rotation angle of the facet joint is 3.28 ± 1.49° in the α angle, -0.26 ± 0.31° in the β angle and 1.84 ± 0.80° in the γ angle.

At 0 and 10 kg, there was a difference in the X axis and γ angle in the left L5-S1 and a difference in the angle of the right L5-S1 in the γ angle, and there was no significant difference in other data (Table 1).

**Table 1:** Displacement and rotation angle of lumbar facet joints in flexion and extension

Displacement Left (mm)	L3-4				L4-5						L5-S1		
	0 kg	10 kg	P		0 kg	10 kg	P			0 kg		10 kg	P
**X**	0.00 ± 1.01	0.28 ± 0.16	0.42		0.45 ± 0.67	0.66 ± 0.59	0.55			1.43 ± 2.79		-0.89 ± 1.21	0.04
**Y**	-0.29 ± 1.14	0.03 ± 0.77	0.30		-0.41 ± 1.02	-0.97 ± 1.12	0.26			-1.55 ± 4.18		0.69 ± 2.23	0.18
**Z**	-4.65 ± 1.99	-2.72 ± 2.11	0.08		-1.89 ± 2.99	-2.51 ± 1.78	0.55			0.81 ± 2.27		0.41 ± 2.13	0.63
**Displacement** **(mm)**													
**Right**													
**X**	-0.12 ± 0.98	0.23 ± 0.19	0.31		0.77 ± 0.97	1.02 ± 0.83	0.64			1.40 ± 2.79		-0.77 ± 1.25	0.06
**Y**	0.19 ± 1.13	0.37 ± 0.66	0.37		0.15 ± 1.60	-0.67 ± 0.45	0.22			-0.51 ± 3.37		-0.33 ± 1.70	0.88
**Z**	-3.20 ± 2.61	-1.26 ± 2.19	0.09		-1.72 ± 3.00	-2.55 ± 1.53	0.45			0.31 ± 1.69		0.92 ± 2.98	0.532
**Angle** **(°)**													
**α**	-6.00 ± 4.49	-3.80 ± 3.62	0.31		-3.51 ± 5.24	-5.96 ± 3.33	0.17			0.97 ± 4.13		3.28 ± 4.73	0.09
**β**	2.30 ± 2.94	1.28 ± 2.16	0.31		0.16 ± 2.06	0.58 ± 1.47	0.67			-0.36 ± 1.74		-0.26 ± 0.99	0.89
**γ**	0.74 ± 1.57	-0.27 ± 1.67	0.16		-0.94 ± 2.82	-0.55 ± 3.24	0.83			-1.12 ± 2.83		1.84 ± 2.53	0.03

## Discussion

The lumbar spine is the most weight-bearing part of the spine, so it is prone to degeneration. Low back pain easily occurs after facet joints degeneration, which seriously affects the quality of life of patients. The kinematics of lumbar facet joints can reflect the motion of facet joints in daily activities, which is helpful for us to analyse the causes of facet joints degeneration. The sitting position is a commonly used posture in daily work and life. At present, there is no related research on the effect of weight bearing on the movement of facet joints in the sitting position.

Previous studies on lumbar facet joint motion are mostly based on the standing position. Song et al. [21] performed related studies on facet joint motion in the standing position and concluded that there was no significant difference in displacement under different weight loads in different segments. Our study found that at 0 kg, the left and right (X axis) and anterior and posterior (Y axis) displacements between L3-4 and L4-5 and the vertical displacement (Z axis) between L3-4 and L4-5 were also smaller than those studied by Song et al., while at 10 kg, the left and right (X axis) and anterior and posterior (Y axis) displacements between L3-4 and L4-5 were relatively smaller than those studied by Song et al., while the vertical displacement (Z axis) was larger. We believe that this difference is due to different postures. When the posture changes from standing to sitting, the pelvis will be further fixed and not easy to move, so when flexion and extension occur, the lumbar vertebrae will compensatively increase the horizontal displacement. When bearing weight, the vertical displacement of the facet joints change more when sitting, which may be related to the change in pelvic position. However, Song et al.‘s study did not measure the rotation angle of facet joints, so it is impossible to compare the rotation angles of facet joints between standing and sitting positions.

Our study mainly observed changes in the displacement distance and rotation angle of facet joints after weight bearing. After loading, in the L3/4 segment, the displacement of the left facet joint in the X axis increased, while that in the Y axis and Z axis decreased, the displacement of the right facet joint in the X axis and Y axis increased, the Z axis displacement decreased, and the rotation angle of the bilateral facet joints decreased. In the L4/5 segment, after loading, the displacement of the Z axis of the left and right sides increases, while the rotation angle of the α angle and β angle increases, while the rotation angle of the γ angle decreases. In the L5/S1 segment, the displacement of the Z axis on the left decreases, the displacement of the Y axis on the right decreases, the displacement of the Z axis increases, the rotation angles of α and γ increase, and the rotation angle of the β decreases. This shows that when the lumbar spine is flexed and extended in the sitting position, the movement of the facet joint is not translation or rotation in one direction but rather compound motion in many directions. After weight-bearing 10 kg, this form of motion is not changed. This is somewhat different from the previous study by Wen et al. [22]. According to the study of Wen et al., when standing, weight bearing will have a certain impact on the horizontal movement of the lumbar spine, but there is little change in the vertical direction. We believe that the difference is a result of the subjects having different intervals under different weight-bearing conditions. Too short intervals will lead to closer contact between the intervertebral disc and the facet joint, which will affect the movement of the facet joint, especially the vertical displacement. Therefore, between different weight-bearing tests, we allowed the subjects to reserve enough rest time so that the joint space of the facet joint could be restored to better study the movement form of the facet joint.

In our study, we also found inconsistencies in left and right motion. For example, in the L3/4 segment, in the flexion and extension of 0 kg, the displacement of the left side is smaller than that of the right side in the X axis, while the displacement of the left side is larger than that of the right side in the Y axis and Z axis. In the case of 10 kg, the displacement of the left side of the Y-axis is smaller than that of the right side, while in the displacement of the Z axis, the right side is smaller than the left side. The asymmetry of this movement also exists in the facet joints of the L4/5 segment and L5/S1. This result is similar to that observed by Kou et al. [23]. It is believed that the inconsistent displacement of the left and right sides is caused by the asymmetry of the articular surface. In our study, we also found asymmetry of the left and right articular surfaces, which led to asymmetry of motion. At the same time, we find that when loading 10 kg, the asymmetry of this kind of motion also exists.

There are still some limitations in the experiment. First, we have a small sample size, only 10 subjects, which may cause errors in the data. Second, we chose younger subjects because older subjects may be more likely to have lumbar facet degeneration, but this may cause age bias. Again, due to the limitations of the DFIS system, we only collected the motion data of L3-S1 facet joints. In future studies, we plan to increase the sample size and measure the normal population of middle-aged and elderly individuals, and we also need to improve the technology to make a wider space for lumbar facet joint collection to collect more motion data of lumbar segments.

Although there are some limitations, we still think that the research is of great significance. The kinematic study of lumbar facet joints can help us find the motion characteristics of lumbar facet joints and then prevent and treat facet joints degeneration. It can also guide the design and assembly of medical instruments such as artificial intervertebral discs. Our study quantified the motion displacement and rotation angle of lumbar facet joints in the sitting position and studied the effect of weight-bearing on lumbar facet joint motion. The study found that weight-bearing does not change the coupling movement of lumbar facet joints, nor does it change the phenomenon of inconsistent movement of left and right facet joints. We hope that through this study, we can improve our understanding of lumbar motion patterns to improve the level of diagnosis and treatment of related diseases.

## Data Availability

The data sets used and/or analysed during the current study are available from the corresponding author on reasonable request.

## Abbreviations

DFIS: Dual fluoroscopic imaging system
ROM: Range of motion
